# Structural Network Analysis Using Diffusion MRI Tractography in Parkinson's Disease and Correlations With Motor Impairment

**DOI:** 10.3389/fneur.2020.00841

**Published:** 2020-09-02

**Authors:** Jelmer G. Kok, Alexander Leemans, Laura K. Teune, Klaus L. Leenders, Martin J. McKeown, Silke Appel-Cresswell, Hubertus P. H. Kremer, Bauke M. de Jong

**Affiliations:** ^1^Department of Neurology, University Medical Center Groningen, University of Groningen, Groningen, Netherlands; ^2^Image Sciences Institute, University Medical Center Utrecht, Utrecht, Netherlands; ^3^Pacific Parkinson's Research Centre, University of British Columbia, Vancouver, BC, Canada

**Keywords:** diffusion weighted imaging (DWI), tractography, network analysis, global and local efficiency, Parkinson's disease, connectivity matrix

## Abstract

Functional impairment of spatially distributed brain regions in Parkinson's disease (PD) suggests changes in integrative and segregative network characteristics, for which novel analysis methods are available. To assess underlying structural network differences between PD patients and controls, we employed MRI T1 gray matter segmentation and diffusion MRI tractography to construct connectivity matrices to compare patients and controls with data originating from two different centers. In the Dutch dataset (Data-NL), 14 PD patients, and 15 healthy controls were analyzed, while 19 patients and 18 controls were included in the Canadian dataset (Data-CA). All subjects underwent T1 and diffusion-weighted MRI. Patients were assessed with Part 3 of the Unified Parkinson's Disease Rating Scale (UPDRS). T1 images were segmented using FreeSurfer, while tractography was performed using ExploreDTI. The regions of interest from the FreeSurfer segmentation were combined with the white matter streamline sets resulting from the tractography, to construct connectivity matrices. From these matrices, both global and local efficiencies were calculated, which were compared between the PD and control groups and related to the UPDRS motor scores. The connectivity matrices showed consistent patterns among the four groups, without significant differences between PD patients and control subjects, either in Data-NL or in Data-CA. In Data-NL, however, global and local efficiencies correlated negatively with UPDRS scores at both the whole-brain and the nodal levels [false discovery rate (FDR) 0.05]. At the nodal level, particularly, the posterior parietal cortex showed a negative correlation between UPDRS and local efficiency, while global efficiency correlated negatively with the UPDRS in the sensorimotor cortex. The spatial patterns of negative correlations between UPDRS and parameters for network efficiency seen in Data-NL suggest subtle structural differences in PD that were below sensitivity thresholds in Data-CA. These correlations are in line with previously described functional differences. The methodological approaches to detect such differences are discussed.

## Introduction

Neuronal degeneration in the substantia nigra resulting in dopamine deficiency in the basal ganglia is a major characteristic pathophysiological change in Parkinson's disease (PD). As the basal ganglia are involved in multiple corticosubcortical networks, PD can be viewed as an extended network disease of the brain (1). In the present study, we aimed to explore structural network differences in PD by combining gray matter (GM) segmentation, diffusion MRI tractography, and complex network analysis in two independent datasets.

The concept that PD symptoms and signs arise from functional impairment in coherent basal ganglia–cortical networks became generally acknowledged after acceptance of the model of segregated circuits (e.g., motor, oculomotor, and limbic), originating from the cortex via the basal ganglia and the thalamus back to particularly frontal cortical regions (2). The prominence of dopamine depletion in the posterior putamen (3) (which is part of the motor circuit in the model) is consistent with the prominent motor features in early PD stages. Further elaborations of the basic model included altered direct and indirect pathways within the basal ganglia (4), leading to an increased basal ganglia output to the thalamus and a subsequent decreased excitatory output back to the cortex. A physiological characteristic of PD-related changes in basal ganglia circuits is a more synchronous firing pattern (4), pointing at reduced segregation of neuronal activities of the basal ganglia loops in PD.

*In vivo* imaging of the human brain has complemented and extended insights gained from animal research and human histological examination of these cerebral pathways, both in health and in disease. Functional imaging with positron emission tomography (PET) using l-[^18^F]fluorodopa has enabled the spatial identification and quantitative assessment of striatal dopamine deficiency, a key feature of PD (5), which is particularly pronounced in posterior parts of the putamen (6, 7). Application of the PET tracer [^18^F]fluorodeoxyglucose (FDG), a marker of regional cerebral metabolism, allowed for identification of a characteristic PD-related pattern of relative decreased metabolic activity in parietal, visual, and lateral premotor and prefrontal cortices and relative increases in the pons, thalamus, pallidum, dorsal putamen, primary motor cortex, and supplementary motor areas (8, 9). Functional interactions between such regions can be identified with resting state fMRI and targeted activation paradigms. For example, resting state fMRI enabled the demonstration of reduced functional connectivity between the posterior putamen and inferior parietal cortex together with increased anterior putamen–inferior parietal coupling, a remapping considered to reflect reduced spatial segregation between different corticostriatal loops in PD (10). By applying an fMRI visual optic-flow paradigm, mimicking the perception of forward locomotion, we previously found that interruption of such a gait-supporting stimulus failed to activate the (pre-)supplementary motor area in PD, while functional connectivity between this region and the visual motion area V5 was enhanced in patients, a result which is consistent with the increased interference of perceptual stimuli with motor intentions in PD (11).

The functional connectivity and inferred interactions between spatially distributed brain regions in fMRI data are based on the temporal correlations of signal changes in such regions, either evoked in hypothesis-driven experiments, or spontaneously occurring (12, 13). A basic network model of small-world architecture, describing the dynamic consequences of local and remote interconnectivity features (14) has strongly encouraged the development of whole-brain “complex network” analysis (15, 16). Applications of the latter in neuroimaging data made it possible to reach higher levels of exploring the brain's neuronal organization that underlies the balance between functional segregation and integration. This coincides with regional modular integration in specialized brain regions as well as global integration based on remote whole-brain interconnections of regional modules. While “connectivity” in the obtained datasets may concern networks of either actual white matter connections or functional associations (13, 15), these networks can generally be mathematically defined as a collection of nodes and edges (links) between pairs of nodes (15). This approach enables the construction of connectivity matrices and the subsequent calculation of various measures, such as global efficiency (E_glob_) and local efficiency (E_loc_) that represent network properties concerning integration and segregation, respectively (16, 17).

In PD, the wide spectrum of specific motor (18) and non-motor (19) symptoms provides a challenge to capture differences in the integrative and segregative properties of the PD-affected cerebral networks. Complex network approaches using resting state fMRI have shown decreased whole brain E_glob_ in PD (20, 21) as well as decreased motor network E_glob_ in preselected cortical and basal ganglia regions (22). E_loc_ differences in distinct nodes have also been reported without differences in average whole-brain E_glob_ and E_loc_ (23). In mildly cognitively impaired PD patients, Pereira et al. demonstrated reduced E_glob_ compared to controls, deriving network parameters from correlation matrices based on (structural) cortical thickness measures (24). While correlation matrices on fMRI data and regional cortical thickness measurements use edges between nodes without taking into account the existence of actual white matter connections, complex network analysis on white matter tracts that interconnect distinct GM regions requires the reliable identification of such structural edges, e.g., based on diffusion MRI tractography. With diffusion MRI, E_glob_ reduction has been shown in PD (25–27). The number of complex network studies that have been published on PD-associated differences in structural cerebral connectivity however remains limited, in such a way that it remains difficult to draw unequivocal conclusions concerning changes in specific networks (28).

In the present PD imaging study, we performed complex network analysis on structural white matter connectivity. This required the initial steps of (i) GM segmentation to provide regions of interest (ROIs) and (ii) diffusion MR tractography rendering streamlines. From these data, connectivity matrices were constructed serving the model of nodes and edges, to be used in subsequent calculations. This was performed in two independent datasets, each consisting of PD patients and control subjects. We hypothesized finding reductions of both E_glob_ and E_loc_ in PD patients and negative correlations of these measures with clinical motor scores in interconnected brain regions previously implicated in functionally impaired networks in PD.

## Materials and Methods

Two MRI datasets of PD patients and controls were acquired. One dataset [Dutch dataset (“Data-NL”)] was acquired at the University Medical Center Groningen, the Netherlands. The other [Canadian dataset (“Data-CA”)] was acquired at the Pacific Parkinson's Research Center of the University of British Columbia, Vancouver, Canada. Unless specified otherwise, the same procedures were followed with both datasets.

### Participants

#### Data-NL

For the first dataset, 15 PD patients and 16 healthy controls (HCs) were included. During processing of the imaging data, two subjects had to be excluded from further analysis due to suboptimal quality of the data, leaving 14 patients and 15 age-matched controls for the final analyses. PD patients fulfilled the UK Brain Bank criteria for PD (29, 30). HCs were required not to have first-degree family members with parkinsonism or dementia. All subjects underwent MRI scanning. Before MRI, antiparkinson medication and benzodiazepines were withheld for at least 12 and 24 h, respectively. Part 3 of the Unified Parkinson's Disease Rating Scale (UPDRS) was applied in the PD group. Table 1 shows some demographics of both datasets.

**Table 1:** Demographics of both datasets.

	**Dataset NL**		**Dataset CA**	
	**PD**	**HC**	**PD**	**HC**
Number	14	15	19	18
Gender (M/F)	10/4	10/5	12/7	4/14
Age (years)	65.0 (7.1)	61.4 (7.8)	60.7 (7.8)	56.9 (6.5)
UPDRS motor score	18.4 (6.2)	N/A	25.4 (14.0)	N/A
Symptom duration (years)	5.2 (3.7)	N/A	4.8 (2.8)	N/A

*mean (+/– std) unless otherwise specified*.

*PD, Parkinsons disease; HC, healthy control*.

#### Data-CA

In Data-CA, 19 subjects with PD and 18 age-matched HCs were enrolled. All PD patients were diagnosed and under the care of a tertiary-care movement disorders specialist. Exclusion criteria included atypical parkinsonism, other neurological or psychiatric conditions, and use of antidepressants, hypnotics, or dopamine blocking agents. In this dataset, part 3 of the UPDRS was applied in the PD group as well. Both motor assessment and image acquisition were done after withdrawal of l-DOPA for 12 h and dopamine agonists for 18 h.

### Image Acquisition

#### Data-NL

Subjects were scanned using a Philips Intera 3.0-T scanner (Philips, Best, the Netherlands) with an eight-channel head coil. T1-weighted images were acquired using a turbo field echo pulse sequence with the following parameters: 170 axial slices of 1 mm without gap, field of view (FOV) (ap × rl × fh) 232 × 256 × 170 mm^3^, acquisition matrix (ap × rl) 231 × 256, reconstructed voxel size 1 × 1 × 1 mm^3^, TR 9 ms, TE 3.5–3.57 ms, and flip angle 8° for a total scan duration of 251 s. Diffusion-weighted imaging (DWI) was performed using a single-shot spin-echo echo-planar imaging sequence with the following parameters: 65 axial slices of 2 mm without gap, FOV 240 × 240 × 130 mm^3^, acquisition matrix 117 × 120, reconstructed voxel size 1.88 × 1.88 × 2 mm^3^, TR 10,833–11,053 ms, TE 113 ms, flip angle 90°, 60 diffusion directions (and seven non-diffusion-weighted scans averaged to one volume), and *b*-value 4,000 s/mm^2^, for a total scan duration of 748–763 s.

#### Data-CA

Subjects were scanned using a Philips Achieva 3.0-T scanner with an eight-channel head coil. T1-weighted images were acquired using a turbo field echo pulse sequence with the following parameters: 170 axial slices of 1 mm without gap, FOV 256 × 200 × 170 mm^3^, acquisition matrix 256 × 200, voxel size 1 × 1 × 1 mm^3^, TR 7.569–7.746 ms, TE 3.53–3.59 ms, and flip angle 8° for a total scan duration of 394 s. Diffusion-weighted images were acquired using a single-shot spin-echo echo-planar imaging sequence with the following parameters: 60 axial slices of 2.2 mm without gap, FOV 212 × 212 × 132 mm^3^, acquisition matrix 95 × 96, reconstructed voxel size 0.83 × 0.83 × 2.2 mm^3^, TR 6,053–6,898 ms, TE 69 ms, flip angle 90°, 32 diffusion directions (and one non-diffusion-weighted scan), and *b*-value 700 s/mm^2^, for a total scan duration of 212–248 s. Three repetitions of the diffusion-weighted acquisition were performed.

### Image Processing

**Figure 1:**
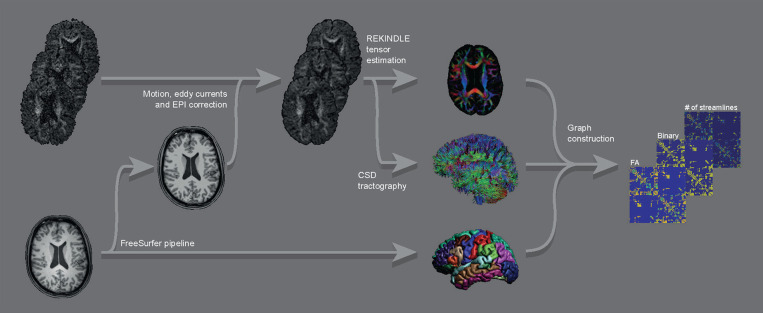
Graphical overview of the main parts of the image processing pipeline for a single subject. Raw DWI images (top left) were corrected for motion, eddy currents, and EPI distortions, followed by constructing FA maps and streamline sets. These were combined with the ROIs from the FreeSurfer output of the raw T1 image (bottom left) into graphs (binary and weighted by FA and by number of streamlines).

First, both T1-weighted and diffusion-weighted images were processed to render the ROIs and the streamlines, which were subsequently combined to form the connectivity matrices. From these matrices, E_glob_ and E_loc_ were calculated. See Figure 1 for a graphical overview of the image processing pipeline.

#### GM Segmentation

For GM segmentation (providing nodes for the complex network analysis), the T1 images were processed using the FreeSurfer pipeline (31) (RRID:SCR_001847), which included defining ROIs according to the Desikan–Killiany atlas (32). FreeSurfer version 5.3 was applied on a cluster of processors running Linux (Ubuntu 12.04.5 LTS, CPU model AMD Opteron Processor 6272). Default parameters were used. The output of every subject was checked visually by viewing the subcortical segmentation and the white and pial surfaces overlaid on coronal, sagittal, and axial T1 slices. If necessary, adjustments were made to the output followed by rerunning parts of the FreeSurfer pipeline. As a result, data from all subjects were suitable for further analysis. The resulting files containing the ROIs (the aparc+aseg.mgz files) and the T1 volumes (T1.mgz files) were converted to the nifti file format and stored for this purpose. It should be noted that using these methods, the ROIs were in the individual subject space, reducing the need for non-linear spatial transformations of the diffusion-weighted images.

In the ROI file, ROIs that were of no interest, for example, the ventricles and the cerebellum (which was not fully covered in all acquisitions), were excluded from the analysis, resulting in 85 ROIs for each subject. These ROIs comprised all cortical ROIs from the Desikan–Killiany atlas (33) as well as the thalamus proper, caudate, putamen, pallidum, hippocampus, amygdala, accumbens area and ventral DC (all bilateral), and brainstem.

The putamina were separated by the vertical plane traversing the anterior commissure in an anterior part and a posterior part, taking into account that the posterior part is more severely affected in PD (3, 6, 7). This was achieved by (a) applying the talairach.xfm transform as saved by the FreeSurfer output to the T1.mgz file (i.e., resulting in the anterior–posterior direction of the voxels in this new file being parallel to the anterior–posterior direction of the brain), (b) using this file to construct a plane through the anterior commissure and orthogonal to the anterior commissure–posterior commissure line with a thickness of 2 mm, (c) applying the inverse of the talairach.xfm to this plane, and (d) separating the putamina in native space according to this plane.

#### White Matter Processing

The white matter analyses were performed using software running under MATLAB, MathWorks, Inc., Natick, Massachusetts, USA. In order to define the streamlines (providing edges for the complex network analysis) for each subject, the diffusion-weighted images were corrected for motion, eddy currents, and echo planar imaging (EPI) distortions, followed by tractography (see pipeline below). The DWI data were processed in ExploreDTI (34) (RRID:SCR_001643) running under Linux (Ubuntu 10.04 LTS, CPU model Intel Core i7-2600). The pipeline consisted of the following steps:

1. To aid in later registration, the FreeSurfer T1 nifti files were masked using ExploreDTI (and checked visually afterwards), applying a kernel size of morphological operators of 5 and a threshold of 0.05.
2. For each subject in Data-CA, the three DWI sessions were concatenated resulting in one file with three *b* = 0 s/mm^2^ volumes and 96 *b* = 700 s/mm^2^ volumes.
3. Diffusion gradient directions were corrected for the image angulation as described in the Philips PAR header.
4. The DWI volumes of all subjects were corrected for motion, eddy currents, and EPI distortions using default parameters (33, 35–37). As a part of this, the (originally distorted) DWI volumes were aligned with the masked T1 file.
5. Tensor estimation was performed using the REKINDLE method (38).
6. The processed diffusion data were checked visually for each subject by (a) viewing all three planes of the corrected diffusion-weighted images in a movie loop, (b) viewing the axial slices of the color-coded fractional anisotropy (FA) map, and (c) viewing all three planes of the color-coded FA map overlaid on the T1 volume. Data-CA did not raise concerns in this regard, but Data-NL showed many erroneous motion correction results, due to the high *b*-value employed (4,000) and the accompanying low SNR. Therefore, the correction process for this dataset was rerun using adjusted motion correction settings. The number of histogram bins was set to 16 and the number of data samples to 8,000. Also, the “Scales” parameters for the Elastix program were adjusted so that rotation, scaling, and shearing were constrained (set to 10^6^, whereas the Scales parameters for translation were kept at 1). After rerunning all subjects' scans with these settings and using the quality assurance program as described above, all datasets were suitable for further analysis, except for two datasets which were excluded as the correction results remained poor.
7. Constrained spherical deconvolution (CSD) whole-brain tractography (39–41) was performed using the following parameters: step size 1 mm, fiber orientation distribution (FOD) threshold 0.1, angle threshold 30°, length range 50–500 mm, and seed point resolution 2 × 2 × 2 mm. The maximum harmonic order was set to 8 for Data-NL and to 4 for Data-CA. This resulted in one set of streamlines for each subject.

#### Complex Network Analysis

Connectivity matrices were created for each subject by combining the ROIs (nodes) and the streamlines (edges). The matrices were constructed with 87 rows and 87 columns (for 87 ROIs) in which the intersection of two ROIs was labeled by the mean FA if at least one streamline was found between these ROIs. Zeros were placed both at intersections without a streamline and on the main diagonal. Similarly, connectivity matrices weighted by the number of streamlines (NOS), and binary connectivity matrices (i.e., the distinction between connections present yes or no) were created and used in subsequent analyses.

For the network analyses, an initial threshold was applied by discarding all connections present in <50% of subjects (42). That is, for every possible connection out of a total of 87 × 86 = 7,482 connections, only those connections that were found using tractography in at least 50% of all subjects would be used in subsequent analyses. In this way, we aimed to reach a balance between the number of false-positive streamlines (also called spurious streamlines, i.e., following trajectories without the presence of actual underlying fibers) and false-negative streamlines (i.e., underlying trajectories not being found by the streamline algorithm). This was done independently for each dataset. Next, the FA weighted connectivity matrices were normalized per dataset; i.e., all FA values in these matrices were divided by the maximum value of all FA matrices within the corresponding dataset. The same was done with the NOS matrices. This resulted in values between 0 and 1, which is a prerequisite for the subsequent complex network analysis, while at the same time, relative differences between subjects remained. The E_glob_ and E_loc_ were calculated using the Brain Connectivity Toolbox that accompanied a 2010 paper by Rubinov and Sporns (16). E_loc_ was calculated using the efficiency_bin (for binary matrices) and efficiency_wei (for weighted matrices) functions from the toolbox. E_glob_ was calculated using the formula presented in Appendix A of the paper; custom code and the distance_wei function from the toolbox were used. For each connectivity matrix (of each subject), E_glob_ and E_loc_ were calculated across the entire brain, providing whole-brain values. Whole-brain E_glob_ and E_loc_ were constructed by taking the mean of the nodal values of E_glob_ and E_loc_, respectively. Nodal values for E_glob_ and E_loc_ were also stored separately.

### Statistical Analysis

Within each dataset and for the different weightings separately, whole-brain E_glob_ and whole-brain E_loc_ values were compared between PD patients and HCs using a Student *t*-test (alpha 0.05) and related to UPDRS motor scores within the PD group using Pearson's correlation. The same analyses were performed for nodal E_glob_ and nodal E_loc_ and then for each node separately. Here, a false discovery rate (FDR) of 0.05 was applied to correct for multiple comparisons, for each measure and weighting separately.

## Results

**Figure 2:**
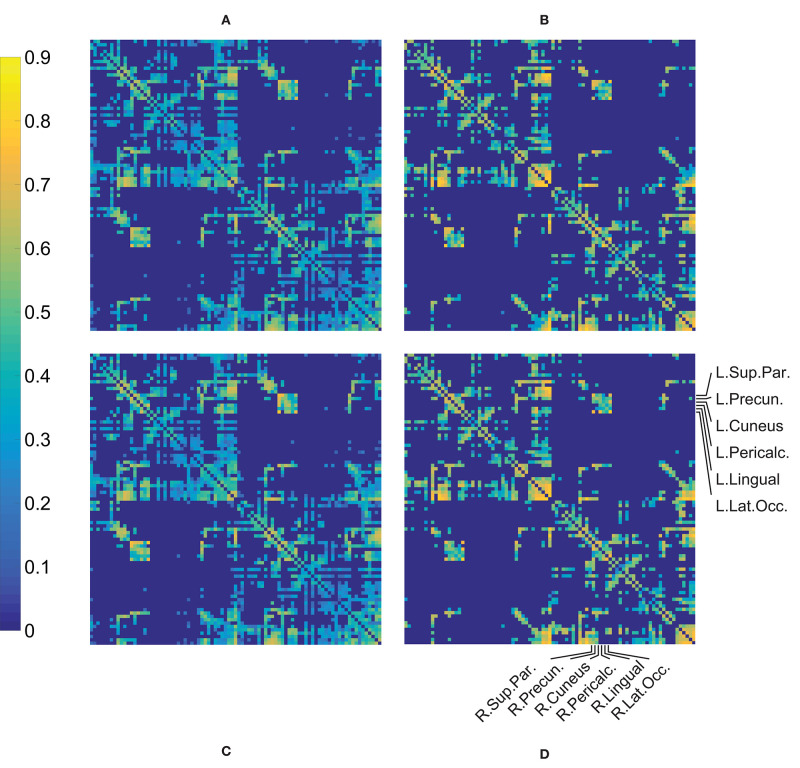
Mean of the FA weighted connectivity matrices per group. The processed individual matrices were used (i.e., thresholded and normalized, see text) in constructing these mean matrices. **(A)** HC Data-NL, **(B)** patient Data-NL, **(C)** HC Data-CA, **(D)** patient Data-CA. Supplementary Figure 1 shows Figure 1D but includes the names of all (87) ROIs.

A qualitative assessment of the FA weighted connectivity matrices revealed a clear consistency between the patterns of the four groups (Figure 2). These matrices were constructed after thresholding and normalization (during which the Data-NL FA values were divided by 0.666 and the Data-CA values by 0.661, which were the respective maxima of the corresponding datasets) and averaging per group. The similarity is illustrated by the robust cross-callosal connections between left and right occipital regions (via the splenium) and between left and right (pre)motor areas in the two datasets. Furthermore, the mean FA per connection was distributed similarly in both datasets (higher pixel values show at similar locations). Also see Supplementary Figure 1 which can be enlarged, in such a way that the labels of all separate ROIs can be read, allowing for a more detailed assessment of the connectivity matrices.

### Data-NL

**Figure 3:**
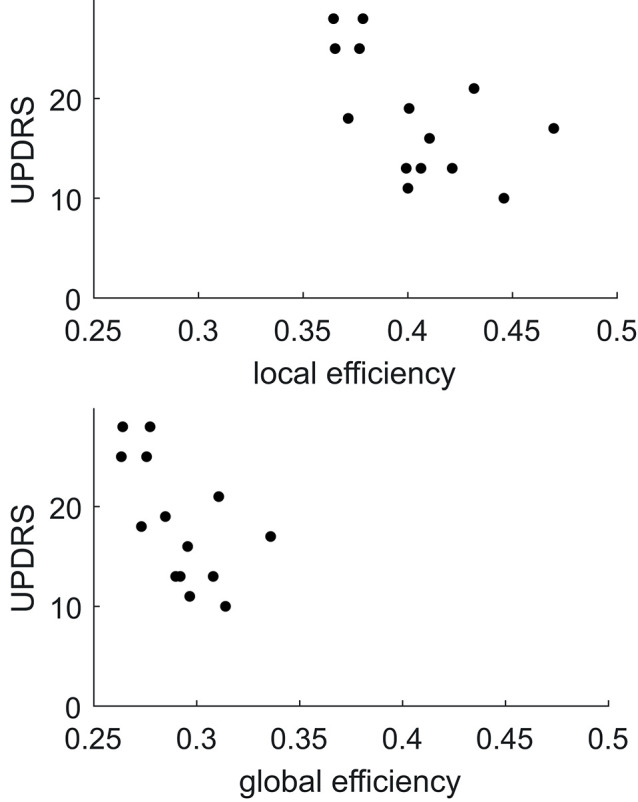
Plots of local and global efficiency calculated from the FA weighted connectivity matrices vs. the UPDRS score in PD patients of Data-NL.

**Figure 4:**
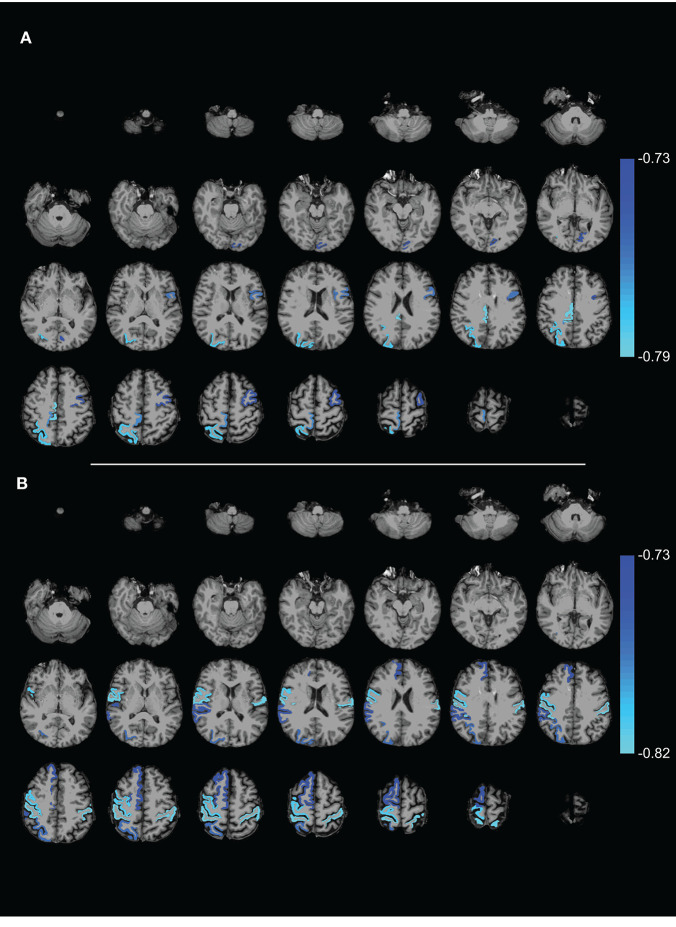
Axial slices showing ROIs with **(A)** local efficiency and **(B)** global efficiency that (negatively) correlated with the UPDRS motor score (FDR 0.05). The complex network measures were calculated from the FA weighted connectivity matrices in PD patients of Data-NL. The scale on the color bar represents Pearson's linear correlation coefficient.

Comparing FA weighted network parameters between the PD and HC groups in Data-NL [mean (std)], whole-brain E_glob_ [PD 0.29 (0.02); HC 0.29 (0.02)], and whole-brain E_loc_ [PD 0.40 (0.03); HC 0.40 (0.03)] did not yield significant differences. Neither were such differences found at regional levels when comparing the nodal E_glob_ and nodal E_loc_ values. However, we did see significant correlations between network measures and clinical PD parameters. Whole-brain E_glob_ and E_loc_ negatively correlated with the UPDRS motor score (E_glob_ *p* = 0.02 and E_loc_ *p* = 0.03), meaning that lower efficiency scores accompanied worse motor symptoms (see Figure 3). At the nodal level, significant negative correlations (using an FDR of 0.05) between E_glob_ and E_loc_ with the UPDRS motor score resulted in two distinct patterns of regional cortical involvement: particularly, the right superior parietal cortex and right posterior cingulate cortex showed strong negative correlations between the UPDRS motor score and E_loc_, while for E_glob_, the strongest negative correlation with the UPDRS motor score concerned the sensorimotor cortex (Figure 4). At more lenient FDR values (e.g., up to 0.2), the number of regions in these patterns considerably increased, with an overlap between the two patterns. Analysis of the NOS and binary matrices did not result in any significant differences or correlations.

### Data-CA

Similar to the analysis in Data-NL, in all weightings (FA, NOS, and binary), none of the network measures showed significant differences between PD and HC in Data-CA [comparing FA weighted network parameters: mean (std) E_glob_ PD 0.34 (0.02)/HC 0.33 (0.03) and E_loc_ 0.55 (0.03)/0.54 (0.04)]. In contrast to the findings in the Data-NL set, however, no significant correlations between any of the measures and the UPDRS motor score were found in this dataset.

## Discussion

The complex network analysis on connectivity matrices constructed using GM segmentation and DWI tractography did not result in significant differences when comparing the PD patients and HC, either in Data-NL or in Data-CA. This suggests that PD did not lead to obvious changes in anatomical interconnection. However, while the connectivity matrices of the four groups indeed showed similar patterns, the negative correlations between both E_glob_ and E_loc_ and the UPDRS motor score in PD of Data-NL (at whole-brain and nodal levels) point to subtle disease-related reductions of integrative and segregative properties of the brain's structural network in PD. To possibly explain why these correlations were identified only in Data-NL and not in Data-CA, clinical explanations and methodological issues will be addressed.

### Clinical Neuroscience Considerations

No differences were found between patients and HCs, whereas decreases of efficiency values have previously been shown with fMRI (20–22) and structural MRI (24–26). At the whole-brain level, differences in E_glob_ and E_loc_ provide a general indication of disease-associated differences in the integration and segregation capacities of the entire cerebral network. At the nodal level, E_glob_ and E_loc_ differences may provide insight into the changed contributions of specific brain regions to functional integration and regional specialization. It should be considered that, while structural network differences may be subtle in PD, functional network differences identified with fMRI may be more robust, preceding structural changes.

We did, however, find that E_glob_/E_loc_ of structural interconnections correlated negatively with UPDRS scores in PD, which is generally consistent with a decrease of efficiency values in previous PD studies. The observed PD-related effect on posterior parietal nodal level E_loc_ suggests impaired efficiency of posterior parietal processing in PD, which is consistent with the enhanced impact of visual cues on motor actions (43, 44), including gait (45). It has been argued that reduced dorsal visual pathway function due to less elaborate early-stage visuomotor processing results in functional shortcuts to remote mediofrontal motor regions (11). The impact of PD pathology on posterior cortical regions can also be inferred from parietal atrophy (46) and the PD-related profile of cerebral metabolism which is characterized by reduced parietal metabolism (8, 9). With structural connectome-based analysis, reduced E_loc_ in particularly parietal regions were very recently identified in a large PD patient group (*n* = 65) compared to 65 matched HCs (47), thus providing support for the observed parietal correlation in our Data-NL patient group. Complementary to the PD-associated reduction of parietal E_loc_, one might speculate that the reduced E_glob_ (at the nodal level) of the motor cortex is based on an increase of non-specific network input from remote regions, in addition to impairment of the specific input from basal ganglia/thalamus loops and premotor regions. Such a model would fit the previously described decrease of network efficiency centered onto the motor cortex (20, 22, 48) together with relatively increased metabolism at this motor target site (8, 9).

### Methodological Considerations

In Data-NL, we identified negative correlations between the above-described parameters for network efficiency and UPDRS motor scores, which was not found in Data-CA. An important difference in data acquisition between the two groups concerned the employed *b*-value, which was higher in Data-NL (4,000), leading to a lower SNR than in Data-CA (*b*-value 700). In Data-NL, this resulted in suboptimal tensor estimations and accompanying FA values which tended to be lower (illustrated in Figure 2). Still, CSD tractography benefits from this *b*-value together with 60 diffusion gradient directions, compared to the more usual *b*-value of around 1,000 and fewer diffusion gradient directions, especially when it comes to resolving fiber crossings (49). The *b*-value of 700 in Data-CA thus assured FA estimations to be more reliable; however, together with the limited number (32) of diffusion directions (and the accompanying maximum harmonic order of 4), it does not serve the best interests of CSD. For example, crossing fibers are better separable at a maximum harmonic order of 8. As a result, more connections were found in Data-NL reflected by more dense connectivity matrices (see Figure 2). Ideally, probably an intermediate *b*-value (or multiple *b*-values) and at least 60 diffusion gradients should be employed. Nonetheless, the patterns in the connectivity matrices were very similar between datasets, suggesting that these matrices may be constructed robustly, despite the applied (very different) acquisition parameters, and the adjustments in the motion correction tuning parameters (i.e., restricting the rotation, sharing, and scaling) that had to be applied in Data-NL.

Assessing tracts between cerebral regions implies demarcation of such regions. In the present study, the latter was performed by defining ROIs according to the Desikan–Killiany atlas (32). It may be evident that improvement in defining nodes and edges is a topic of further development, although to date, no simple solutions exist. Whereas ideally nodes and edges would form a complete (i.e., connections at all locations of underlying biological connections and nowhere else), weighted (i.e., connectivity measures correlating with “connection strength”), and signed (i.e., reflecting the direction of information processing) connectivity matrix, we are far from quantifying the brain's network in this way when constructing diffusion MRI tractography-based connectivity matrices (50–52). Several ways of defining the nodes are available, for example, based on atlases, randomness, functional information, and voxel (50), each with its own strengths and weaknesses. Still, the presently applied Desikan–Killiany atlas has proven to be successful in the past, e.g., (53). Furthermore, we chose to use the FA and NOS weighted and binary (i.e., unweighted) connectivity matrices. Next to the problem of false-negative and false-positive streamlines, there are a number of other methodological considerations on why these may not be suitable for quantifying “connectivity health” (51). In general, we are looking at streamlines drawn in the diffusion field rather than tracking the actual fibers (albeit with the assumption that they somehow correlate) and at best infer indirect measures of the integrity of these underlying fibers. The impossibility of showing differences using these techniques does not mean that integration and segregation functions of the brain do not differ between groups. However, to date, the weightings we employed are the most widely used way of weighting the connectivity matrices. Also, in comparable analyses, intrasubject variability has been shown to be lower than intersubject variability, suggesting that biological processes are being measured (54).

A general issue to consider with regard to (i) the absence of differences when comparing PD and HC and (ii) the observed anticorrelation between network efficiency parameters in only Data-NL and not in Data-CA is the fact that PD is a heterogeneous disease, not only with regard to the spectrum of symptoms but also regarding disease progression (18, 19, 55). Together with the limited number of subjects, disease heterogeneity may have compromised sensitivity to find differences within and between the datasets. On the other hand, one might argue that PD patients and controls did indeed not differ with respect to the integrative and segregative properties of their brains' structural networks. In this respect, it should be kept in mind that functional changes may likely develop before the onset of structural network changes, which might contribute to the discrepancy between previous functional studies and our structural graph analyses. Including larger patient numbers and more severely affected patients has recently shown to yield more robust results (47). The particular strengths of the present study are (i) the inclusion of two independent datasets, each obtained with a different acquisition scheme, and (ii) the use of CSD tractography, which better enables resolving crossing fibers than DTI tractography and thus results in less false-negative streamlines.

## Conclusion

From a qualitative point of view, the connection matrices among the groups in two data sets showed a general similarity, which reinforces the confidence in the results of subsequent quantitative analyses. The negative correlation between E_glob_/E_loc_ and the UPDRS scores in Data-NL suggested specific PD-related network differences for posterior parietal and sensorimotor cortical regions. Particularly, the observed parietal effect reinforces current reports on the involvement of this posterior brain region in PD. In this way, the present study provides leads to extend the search for biomarkers in PD using diffusion MRI complex network analysis. This includes the need to study longitudinal data, increase the sample size, and optimize acquisition parameters. Moreover, analyses need further improvements and validation before certainty can be reached about reduced integrative and segregative capacities of the brain affected by PD.

## Data Availability Statement

The datasets generated for this study are available on request to the corresponding author.

## Ethics Statement

The studies involving human participants were reviewed and approved by the Ethics Committee of the University Medical Center Groningen and the University of British Columbia Ethics Board. The patients/participants provided their written informed consent to participate in this study.

## Author Contributions

MM and BJ: conceptualization. LT: data collection. JK and AL: methodology and software. JK: data curation, formal analysis, and visualization. JK, HK, and BJ: validation and writing and editing the manuscript. AL, LT, KL, MM, and SA-C: editing the manuscript. BJ and HK: supervision. HK: funding acquisition. All authors: contributed to the article and approved the submitted version.

## Conflict of Interest

The authors declare that the research was conducted in the absence of any commercial or financial relationships that could be construed as a potential conflict of interest.

## References

[1] Galvan A Wichmann T. Pathophysiology of parkinsonism. Clin Neurophysiol. (2008) 119:1459–74. 10.1016/j.clinph.2008.03.017 18467168 PMC2467461

[2] Alexander GE DeLong MR Strick PL. Parallel organization of functionally segregated circuits linking basal ganglia and cortex. Ann Rev Neurosci. (1986) 9:357–81. 10.1146/annurev.ne.09.030186.002041 3085570

[3] Kish SJ Shannak K Hornykiewicz O. Uneven pattern of dopamine loss in the striatum of patients with idiopathic Parkinson's disease. N Engl J Med. (1988) 318:876–80. 10.1056/NEJM198804073181402 3352672

[4] Wichmann T DeLong MR Guridi J Obeso JA. Milestones in research on the pathophysiology of Parkinson's disease. Mov Disord. (2011) 26:1032–41. 10.1002/mds.23695 21626548 PMC4272856

[5] Leenders KL Palmer AJ Quinn N Clark JC Firnau G Garnett ES. Brain dopamine metabolism in patients with Parkinson's disease measured with positron emission tomography. J Neurol Neurosurg Psychiatry. (1986) 49:853–60. 10.1136/jnnp.49.8.853 3091770 PMC1028944

[6] Nurmi E Ruottinen HM Bergman J Haaparanta M Solin O Sonninen P. Rate of progression in Parkinson's disease: A 6-[18F] fluoro-L-dopa PET study. Mov Disord. (2001) 16:608–15. 10.1002/mds.1139 11481683

[7] Guttman M Burkholder J Kish SJ Hussey D Wilson A DaSilva J. [11C] RTI-32 PET studies of the dopamine transporter in early dopa-naive Parkinson's disease implications for the symptomatic threshold. Neurology. (1997) 48:1578–83. 10.1212/WNL.48.6.1578 9191769

[8] Eidelberg D. Metabolic brain networks in neurodegenerative disorders: a functional imaging approach. Trends Neurosci. (2009) 32:548–57. 10.1016/j.tins.2009.06.003 19765835 PMC2782537

[9] Teune LK Renken RJ Mudali D de Jong BM Dierckx RA Roerdink JB. Validation of parkinsonian disease-related metabolic brain patterns. Mov Disord. (2013) 28:547–51. 10.1002/mds.25361 23483593

[10] Helmich RC Derikx LC Bakker M Scheeringa R Bloem BR Toni I. Spatial remapping of cortico-striatal connectivity in Parkinson's disease. Cerebral Cortex. (2010) 20:1175–86. 10.1093/cercor/bhp178 19710357

[11] van der Hoorn A Renken RJ Leenders KL de Jong BM. Parkinson-related changes of activation in visuomotor brain regions during perceived forward self-motion. PLoS ONE. (2014) 9:e95861. 10.1371/journal.pone.0095861 24755754 PMC3995937

[12] Biswal B Zerrin Yetkin F Haughton VM Hyde JS. Functional connectivity in the motor cortex of resting human brain using echo-planar mri. Mag Reson Med. (1995) 34:537–41. 10.1002/mrm.1910340409 8524021

[13] Friston KJ Functional and effective connectivity in neuroimaging: a synthesis. Human Brain Mapp. (1994) 2:56–78. 10.1002/hbm.460020107

[14] Watts DJ Strogatz SH. Collective dynamics of “small-world” networks. Nature. (1998) 393:440–2. 10.1038/30918 9623998

[15] Bullmore E Sporns O. Complex brain networks: graph theoretical analysis of structural and functional systems. Nat Rev. (2009) 10:186–98. 10.1038/nrn2575 19190637

[16] Rubinov M Sporns O. Complex network measures of brain connectivity: uses and interpretations. Neuroimage. (2010) 52:1059–69. 10.1016/j.neuroimage.2009.10.003 19819337

[17] Latora V Marchiori M. Efficient behavior of small-world networks. Phys Rev Lett. (2001) 87:198701. 10.1103/PhysRevLett.87.198701 11690461

[18] Jankovic J. Parkinson's disease: clinical features and diagnosis. J Neurol Neurosurg Psychiatry. (2008) 79:368–76. 10.1136/jnnp.2007.131045 18344392

[19] Sauerbier A Jenner P Todorova A Chaudhuri KR Non motor subtypes and Parkinson's disease. Parkinsonism Related Disord. (2016) 22:S41–6. 10.1016/j.parkreldis.2015.09.027 26459660

[20] Skidmore F Korenkevych D Liu Y He G Bullmore E Pardalos PM. Connectivity brain networks based on wavelet correlation analysis in Parkinson fMRI data. Neurosci Lett. (2011) 499:47–51. 10.1016/j.neulet.2011.05.030 21624430

[21] Göttlich M Münte TF Heldmann M Kasten M Hagenah J Krämer UM. Altered resting state brain networks in Parkinson's disease. PLoS ONE. (2013) 8:e77336. 10.1371/journal.pone.0077336 24204812 PMC3810472

[22] Wei L Zhang J Long Z Wu G-R Hu X Zhang Y. Reduced topological efficiency in cortical-basal ganglia motor network of Parkinson's disease: a resting state fMRI study. PLoS ONE. (2014) 9:e108124. 10.1371/journal.pone.0108124 25279557 PMC4184784

[23] Berman BD Smucny J Wylie KP Shelton E Kronberg E Leehey M. Levodopa modulates small-world architecture of functional brain networks in Parkinson's disease. Mov Disord. (2016) 31:1676–84. 10.1002/mds.26713 27461405 PMC5115928

[24] Pereira JB Aarsland D Ginestet CE Lebedev AV Wahlund L-O Simmons A. Aberrant cerebral network topology and mild cognitive impairment in early Parkinson's disease. Human Brain Mapp. (2015) 36:2980–95. 10.1002/hbm.22822 25950288 PMC6869566

[25] Li C Huang B Zhang R Ma Q Yang W Wang L. Impaired topological architecture of brain structural networks in idiopathic Parkinson's disease: a DTI study. Brain Imaging Behav. (2016) 11:113–28. 10.1007/s11682-015-9501-6 26815739

[26] Nigro S Riccelli R Passamonti L Arabia G Morelli M Nisticò R. Characterizing structural neural networks in *de novo* Parkinson disease patients using diffusion tensor imaging. Human Brain Mapp. (2016) 37:4500–10. 10.1002/hbm.23324 27466157 PMC6867369

[27] Vriend C van den Heuvel OA Berendse HW van der Werf YD Douw L. Global and subnetwork changes of the structural connectome in *de novo* Parkinson's disease. Neuroscience. (2018) 386:295–308. 10.1016/j.neuroscience.2018.06.050 30004009

[28] Atkinson-Clement C Pinto S Eusebio A Coulon O. Diffusion tensor imaging in Parkinson's disease: review and meta-analysis. Neuroimage Clin. (2017) 16:98–110. 10.1016/j.nicl.2017.07.011 28765809 PMC5527156

[29] Litvan I Bhatia KP Burn DJ Goetz CG Lang AE McKeith I. Movement disorders society scientific issues committee report: SIC task force appraisal of clinical diagnostic criteria for parkinsonian disorders. Mov Disord. (2003) 18:467–86. 10.1002/mds.10459 12722160

[30] Hughes AJ Daniel SE Kilford L Lees AJ. Accuracy of clinical diagnosis of idiopathic Parkinson's disease: a clinico-pathological study of 100 cases. J Neurol Neurosurg Psychiatry. (1992) 55:181–4. 10.1136/jnnp.55.3.181 1564476 PMC1014720

[31] Fischl B. FreeSurfer. NeuroImage. (2012) 62:774–81. 10.1016/j.neuroimage.2012.01.021 22248573 PMC3685476

[32] Desikan RS Ségonne F Fischl B Quinn BT Dickerson BC Blacker D. An automated labeling system for subdividing the human cerebral cortex on MRI scans into gyral based regions of interest. Neuroimage. (2006) 31:968–80. 10.1016/j.neuroimage.2006.01.021 16530430

[33] Irfanoglu MO Walker L Sarlls J Marenco S Pierpaoli C. Effects of image distortions originating from susceptibility variations and concomitant fields on diffusion MRI tractography results. Neuroimage. (2012) 61:275–88. 10.1016/j.neuroimage.2012.02.054 22401760 PMC3653420

[34] Leemans A Jeurissen B Sijbers J Jones DK ExploreDTI: a graphical toolbox for processing, analyzing, and visualizing diffusion MR data. In: *17th Annual Meeting of Intl Soc Mag Reson Med* Honolulu (2009). p. 3537.

[35] Klein S Staring M Murphy K Viergever MA Pluim JPW. Elastix: a toolbox for intensity-based medical image registration. IEEE Trans Med imaging. (2010) 29:196–205. 10.1109/TMI.2009.2035616 19923044

[36] Leemans A Jones DK. The B-matrix must be rotated when correcting for subject motion in DTI data. Mag Reson Med. (2009) 61:1336–49. 10.1002/mrm.21890 19319973

[37] Shamonin DP Bron EE Lelieveldt BPF Smits M Klein S Staring M. Fast parallel image registration on CPU and GPU for diagnostic classification of Alzheimer's disease. Front Neuroinformatics. (2014) 7:50. 10.3389/fninf.2013.00050 24474917 PMC3893567

[38] Tax CMW Otte WM Viergever MA Dijkhuizen RM Leemans A. REKINDLE: robust extraction of kurtosis INDices with linear estimation. Mag Reson Med. (2015) 73:794–808. 10.1002/mrm.25165 24687400

[39] Tournier J-D Calamante F Connelly A. Robust determination of the fibre orientation distribution in diffusion MRI: non-negativity constrained super-resolved spherical deconvolution. NeuroImage. (2007) 35:1459–72. 10.1016/j.neuroimage.2007.02.016 17379540

[40] Tax CMW Jeurissen B Vos SB Viergever MA Leemans A. Recursive calibration of the fiber response function for spherical deconvolution of diffusion MRI data. Neuroimage. (2014) 86:67–80. 10.1016/j.neuroimage.2013.07.067 23927905

[41] Jeurissen B Leemans A Jones DK Tournier J-D Sijbers J. Probabilistic fiber tracking using the residual bootstrap with constrained spherical deconvolution. Human Brain Mapp. (2011) 32:461–79. 10.1002/hbm.21032 21319270 PMC6869960

[42] de Reus MA van den Heuvel MP. Estimating false positives and negatives in brain networks. Neuroimage. (2013) 70:402–9. 10.1016/j.neuroimage.2012.12.066 23296185

[43] de Jong BM Frackowiak RSJ Willemsen ATM Paans AMJ. The distribution of cerebral activity related to visuomotor coordination indicating perceptual and executional specialization. Cognitive Brain Res. (1999) 8:45–59. 10.1016/S0926-6410(99)00005-1 10216273

[44] Praamstra P Plat FM. Failed suppression of direct visuomotor activation in Parkinson's disease. J Cognitive Neurosci. (2001) 13:31–43. 10.1162/089892901564153 11224907

[45] van der Hoorn A Hof AL Leenders KL de Jong BM. Narrowing wide-field optic flow affects treadmill gait in left-sided Parkinson's disease. Mov Disord. (2012) 27:580–1. 10.1002/mds.24011 22173937

[46] Meppelink AM de Jong BM Teune LK van Laar T. Regional cortical grey matter loss in Parkinson's disease without dementia is independent from visual hallucinations. Mov Disord. (2011) 26:142–7. 10.1002/mds.23375 20922809

[47] Shah AK Lenka A Saini J Wagle S Naduthota RM Yadav R. Altered brain wiring in Parkinson's disease: a structural connectome based analysis. Brain Connect. (2017) 7:347–56. 10.1089/brain.2017.0506 28595456

[48] Sang L Zhang J Wang L Zhang J Zhang Y Li P. Alteration of brain functional networks in early-stage Parkinson's disease: a resting-state fmri study. PLoS ONE. (2015) 10:e0141815. 10.1371/journal.pone.0141815 26517128 PMC4627652

[49] Jeurissen B Descoteaux M Mori S Leemans A. Diffusion MRI fiber tractography of the brain. NMR Biomed. (2017) 32:e3785. 10.1002/nbm.3785 28945294

[50] Fornito A Zalesky A Breakspear M. Graph analysis of the human connectome: promise, progress, and pitfalls. Neuroimage. (2013) 80:426–44. 10.1016/j.neuroimage.2013.04.087 23643999

[51] Jones DK Challenges and limitations of quantifying brain connectivity *in vivo* with diffusion MRI. Imaging Med. (2010) 2:341–55. 10.2217/iim.10.21

[52] Jones DK Knösche TR Turner R. White matter integrity, fiber count, and other fallacies: the do's and don'ts of diffusion MRI. Neuroimage. (2013) 73:239–54. 10.1016/j.neuroimage.2012.06.081 22846632

[53] Baker STE Lubman DI Yücel M Allen NB Whittle S Fulcher BD. Developmental changes in brain network hub connectivity in late adolescence. J Neurosci. (2015) 35:9078–87. 10.1523/JNEUROSCI.5043-14.2015 26085632 PMC6605159

[54] Bassett DS Brown JA Deshpande V Carlson JM Grafton ST. Conserved and variable architecture of human white matter connectivity. Neuroimage. (2011) 54:1262–79. 10.1016/j.neuroimage.2010.09.006 20850551

[55] Weingarten CP Sundman MH Hickey P Chen N. Neuroimaging of Parkinson's disease: expanding views. Neurosci Biobehav Rev. (2015) 59:16–52. 10.1016/j.neubiorev.2015.09.007 26409344 PMC4763948

