# Supplementary material for: Structural Network Analysis Using Diffusion MRI Tractography in Parkinson's Disease and Correlations With Motor Impairment
Source: Front Neurol. 2020 Sep 2;11:841. doi: 10.3389/fneur.2020.00841 (PMC7492210; doi:10.3389/fneur.2020.00841)
Supplement: Supplementary file 1 [file Image_1.pdf]

Supplementary Material

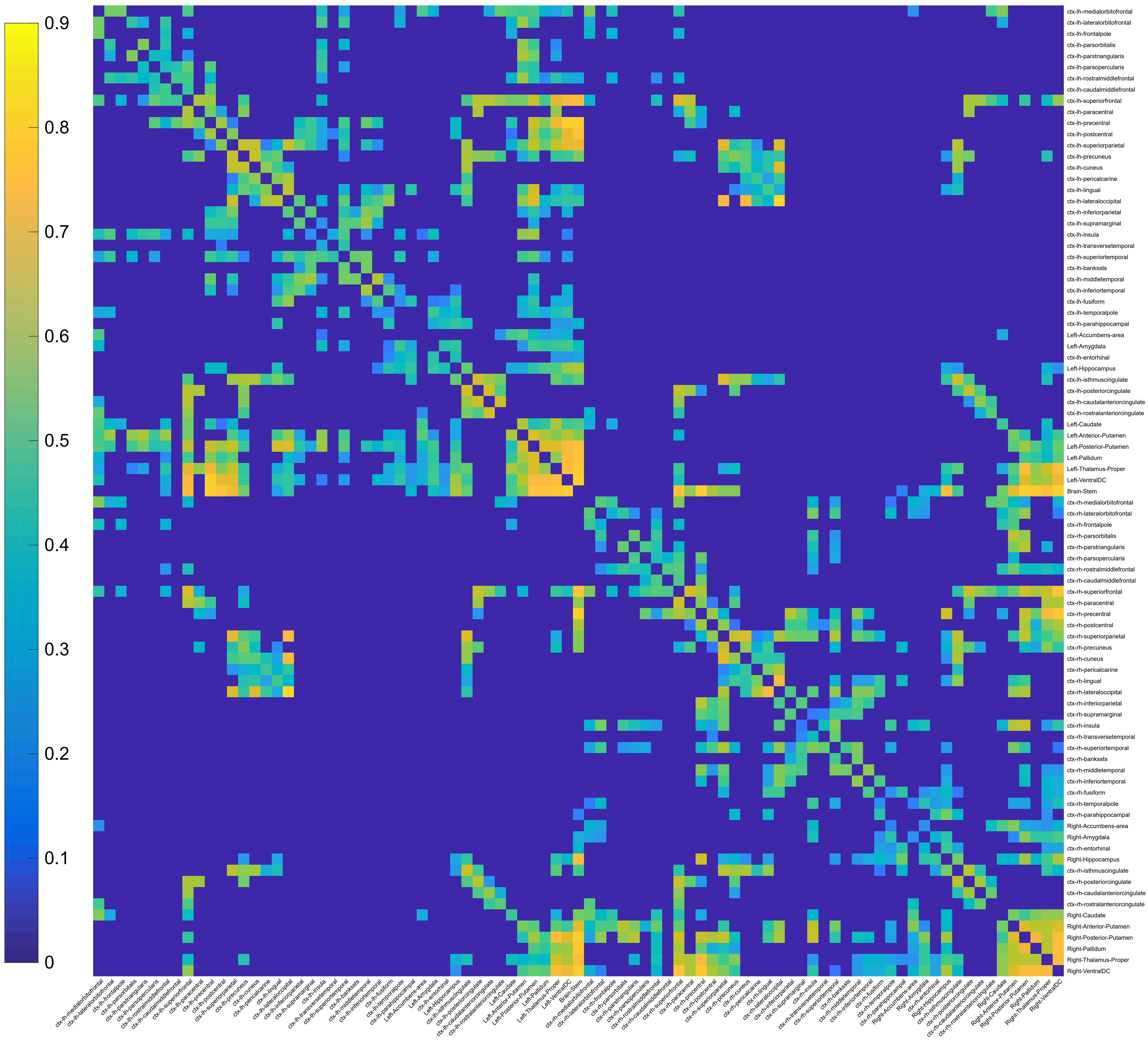

Supplementary Figure 1
Mean of the FA weighted connectivity matrices for the patients in the Canadian dataset. In addition to the otherwise same Figure 1d, this version provides the labels of all separate ROIs, which can be read by enlarging the figure, thus enabling a more detailed assessment of the connectivity matrices in Figure 1.
